# The Value of Routine Intravenous Tranexamic Acid in Total Hip Arthroplasty: A Preliminary Study

**DOI:** 10.1155/2020/2943827

**Published:** 2020-02-11

**Authors:** J. Chin, J. Blackett, D. C. Kieser, C. Frampton, G. Hooper

**Affiliations:** Department of Orthopaedic Surgery and Musculoskeletal Medicine, University of Otago, Christchurch, New Zealand

## Abstract

**Objective:**

To determine the effect on the need for transfusion when intravenous tranexamic acid (TXA) is administered intraoperatively in patients undergoing total hip arthroplasty (THA).

**Method:**

A prospective, double blinded, randomised control trial of 88 patients undergoing THA was randomly allocated to receive 1 g of intravenous TXA or normal saline on induction of anaesthesia. All patients received spinal anaesthesia. The primary outcome measure was transfusion rate, and the secondary outcomes were intraoperative blood loss, haemoglobin levels, length of hospital stay, functional scores, and thromboembolic complications.

**Results:**

19.0% of patients given TXA required a blood transfusion, compared with 20.5% given placebo (*p*=0.87). Secondary outcomes included mean intraoperative blood loss, which was 536.5 ml in the TXA group and 469.8 ml in the placebo group (*p*=0.87). Secondary outcomes included mean intraoperative blood loss, which was 536.5 ml in the TXA group and 469.8 ml in the placebo group (*p*=0.87). Secondary outcomes included mean intraoperative blood loss, which was 536.5 ml in the TXA group and 469.8 ml in the placebo group (*p*=0.87). Secondary outcomes included mean intraoperative blood loss, which was 536.5 ml in the TXA group and 469.8 ml in the placebo group (*p*=0.87). Secondary outcomes included mean intraoperative blood loss, which was 536.5 ml in the TXA group and 469.8 ml in the placebo group (*p*=0.87). Secondary outcomes included mean intraoperative blood loss, which was 536.5 ml in the TXA group and 469.8 ml in the placebo group (

**Conclusions:**

1 g IV TXA administered on induction did not significantly reduce the need for blood transfusion, postoperative blood loss, functional scores, or the length of stay in patients undergoing THA. This trial is registered with ACTRN12610001065088.

## 1. Introduction

As our agingpopulation increases and our arthroplasty volumes grow [1, 2], interest in prophylactic measures to optimise the recovery period of these patients has evolved. One area of interest has targeted a reduction in intraoperative blood loss. This is extremely important in the elderly who have a lower physiological reserve and greater medical comorbidities, thus limiting their ability to tolerate anaemia and haemodynamic instability. Furthermore, blood transfusions are not without risk with complications including pyrexia, allergy, transfusion reaction, and infection transmission [3].

The implementation of enhanced recovery after surgery (ERAS) protocols, including intraoperative blood loss control, has been seen to reduce patient morbidity, hastening recovery, and reduce hospital length of stay [4]. One measure which has gained favour in intraoperative blood loss control is tranexamic acid (TXA) [5].

TXA is an antifibrinolytic agent that potentiates the inhibition of fibrinolysis by inhibiting the activation of plasminogen to plasmin. This reduces clot breakdown and therefore blood loss. TXA has been used for more than 30 years in cardiac surgery where it has shown to reduce postoperative blood loss, transfusion requirements, and the frequency of early surgical revisions for bleeding [6]. It has been more recently applied in orthopaedics, where research has suggested that TXA is an efficacious and cost-effective method of blood conservation [5, 7–9].

However, there remains a theoretical concern that the use of this medication may increase the incidence of thromboembolic events, especially venous thromboembolic events (VTE) such as deep vein thrombosis (DVT) or pulmonary embolism (PE). Furthermore, it is unknown how many patients are needed to be treated with TXA to identify a clinically significant reduction in intraoperative blood loss or to prevent a blood transfusion.

The aim of this study was to accurately assess the effect of 1 g intravenous (IV) TXA, given immediately prior to skin incision, on the requirement for blood transfusion in patients undergoing elective total hip arthroplasty (THA) for osteoarthritis (OA) or osteonecrosis. In addition, intraoperative blood loss, preoperative and postoperative functional scores, and the risk of clinically significant VTE were recorded.

## 2. Methods

We performed a double blind, prospective, randomised control trial (RCT) with patients randomised to receive either 1 g IV TXA or a placebo of IV saline prior to skin incision in patients undergoing elective THA for OA or osteonecrosis.

Patients were recruited by the orthopaedic surgeons of Burwood Hospital, Christchurch, New Zealand.

This RCT only included those patients that received a spinal anaesthetic (+/− general anaesthetic) as part of their procedure. This controls for a confounder in that it is thought a spinal anaesthetic protects against blood loss in total hip arthroplasty by reducing arterial and venous pressure [10].

Patients' consent was attained as was ethical approval from the Central Regional Ethics Committee, Wellington, New Zealand.

Patients were excluded if they had contraindications for TXA (history or risk of thrombosis, active thromboembolic disease, and acquired disturbance of colour vision) or were on clopidogrel, ticagrelor, warfarin, dabigatran, or any other anticoagulant. Patients with renal failure or bleeding disorders were also excluded. Those on aspirin were asked to stop one week prior to surgery. This was recommenced on day 1 postoperation. Those patients that did not receive a single-dose spinal anaesthetic were excluded.

### 2.1. Randomisation and Intervention Delivery

Patients were randomised based on a computer-generated list in a 1 : 1 ratio to TXA or placebo. After the patient was recruited into the study and was in the theatre complex, the randomisation allocation was identified by the unblinded hospital pharmacist who dispensed TXA or placebo in a covered and blinded syringe to the anaesthetist just prior to skin incision.

All patients received a single-dose spinal anaesthetic, with a supplementary general anaesthetic being administered at the discretion of the anaesthetic team.

### 2.2. Procedure and Outcome Measures

THA was performed through a standard posterior approach. Intraoperative blood loss was estimated by the volume in the suction drains and by weighing the swabs, with the assumption that 1 gram was equivalent to 1millilitre of blood. At closure, a single large-bore drain was inserted deep into the fascia lata. Postoperative drainage was recorded for the first 24 hours, and the drains were then removed. Haemoglobin (Hb) was checked preoperatively, one day postoperatively, and four days postoperatively. A blood transfusion threshold of Hb < 80 g/dl was instated. However, patients with symptomatic anaemia with Hb in excess of 80 g/dl were also transfused on clinical reasoning. Time to discharge was recorded from the clinical notes, rounded to the nearest half day.

Functional outcome scores were recorded in clinic both preoperatively and one year postoperatively. These scores included Oxford Hip Score, Western Ontario and McMaster Universities Arthritis Index (WOMAC), and the High Activity Arthroplasty Score (HAAS).

Symptomatic thromboembolic events were screened at follow-up clinics performed at six weeks and variably over the first six months. Any patient who presented to a health practitioner outside of the orthopaedic follow-up clinics over this period was also recorded.

The primary outcomes were the intraoperative blood loss and the transfusion rate, and secondary outcomes were time to discharge, functional outcome scores, and thromboembolic complications.

The outcome measures were compared between randomised groups using Mann–Whitney *U*-tests, and the percentage of patients requiring a transfusion was compared using Fisher's exact test.

To achieve adequate power, with alpha 0.05 and beta 0.80, a total of 200 patients were required to detect a decrease in a transfusion rate of 50% from 0.3 units per patient to 0.15 units per patient.

## 3. Results

**Figure 1:**
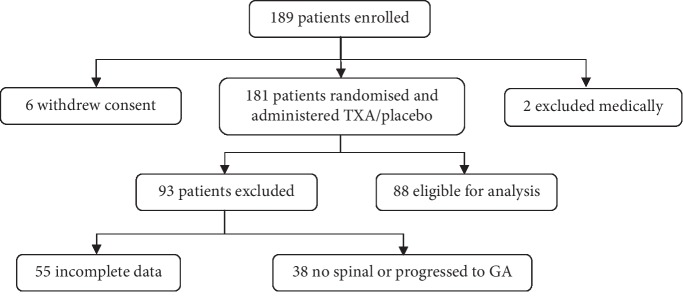
Flowchart of patient inclusion and exclusion.

In total, 189 patients were approached to be recruited for the study, with 88 patients deemed eligible for inclusion. Most patients were excluded following randomisation and administration of intervention/placebo due to progression to general anaesthetic without a spinal anaesthetic or having incomplete data collection (Figure 1.). The mean BMI was 28.9 (18.8 to 41.7).

The total transfusion rate postoperatively, 8 of 42 (19.0%) patients given TXA required a transfusion, compared with 8 of 39 (20.5%) patients given the placebo (*p*=1.00). The patients who were randomised to TXA required 0.34 blood units per patient, compared with 0.44 units per patient (*p*=0.70) in the placebo group (Table 1).

Intraoperative blood loss for patients who received TXA was slightly higher than for those who received placebo (Table 1). Mean total estimated intraoperative blood loss in the TXA group was 536.5 ml and 469.8 ml in placebo (*p*=0.276). Blood loss in swabs was higher in those who received TXA, with a mean of 173.7 g versus 128.8 g (*p*=0.053), respectively. Blood loss measured from the suction showed a mean of 362.8 ml in those who received TXA and 341.1 ml in the placebo group (*p*=0.681). Mean total blood loss recorded in the drain showed blood conservation in those who received TXA compared with placebo, with 560.9 ml and 688.4 ml, respectively (*p*=0.22).

Day 1 haemoglobin levels were higher in those receiving TXA with mean measurements of 108.9 g/l versus 104.3 g/l (*p*=0.114). This was supported by day 4 haemoglobin measures with 105.0 g/l in those who received TXA and 99.8 g/l in those who received placebo (*p*=0.130) (Table 1).

The length of stay did not differ significantly between the two randomised groups (Table 1). The mean length of stay of those who received TXA was 4.3days, compared with 4.8days in those who received placebo (*p*=0.20).

There was no significant difference between TXA and placebo groups in the improvement of functional scales, comparing the preoperative to the one-year postoperative scores. The Oxford Hip Score showed a mean improvement of 25.9 points in those patients who received TXA, compared with 26.7 points in those who received placebo (*p*=0.679). The WOMAC scores were improved by 49.9 points in the TXA group, compared with 50.7 points in the placebo group (*p*=0.864). The mean improvement in the HAAS was 7.5 points in the TXA group, compared with 8.2 points in the placebo group (*p*=0.278).

There was one patient in the TXA group who had a postoperative pulmonary embolism and one patient in the placebo group who had postoperative myocardial infarction.

## 4. Discussion

Our results show that a single dose of 1 g IV TXA prior to incision did not significantly reduce the postoperative blood loss following THA. In addition, no significant differences in transfusion rates were noted.

As observed by the American College of Physicians, “although it is tempting to equate statistical significance with clinical importance, critical readers should avoid this temptation. To be clinically important requires a substantial change in an outcome that matters. Statistically significant changes, however, can be observed with trivial outcomes. And because statistical significance is powerfully influenced by the number of observations, statistically significant changes can be observed with trivial (small) changes in important outcomes. Large studies can be significant without being clinically important, and small studies may be important without being significant [11].”

When one considers the financial cost of such treatment, the total drug cost of tranexamic acid (NZ$58) is less than the production cost of a unit of allogenic blood (NZ$158) [12]. However, using the number needed to treat of 67, this study effectively spent $3886 on TXA to save 1 unit of blood. This cost was not retrieved in a significantly shorter duration of stay as shown by the time to discharge.

However, it may be that 1 g IV TXA is not the optimum dose or the method of administration. Studies have shown that higher doses of TXA and additional routes of administration have a significant effect [5, 8, 9, 13]. In an RCT with 150 patients, Yi and colleagues suggested a single-dose IV TXA plus topical TXA reduce blood loss and the need for transfusion, compared with placebo and IV TXA alone.

Another recent RCT by Xie and colleagues compared blood loss in groups receiving 1 g of IV TXA combined with 2 g of topical TXA, 3 g topical TXA, or 1.5 g IV TXA alone [13]. The mean haemoglobin loss in the combined, local, and IV groups was 2.98 ± 0.78, 3.36 ± 0.78, and 3.89 ± 0.72 g/d, respectively.

Poeran and colleagues [5] performed a retrospective cohort study on over 870,000 patients in the United States, which showed that patients who received TXA intraoperatively had lower rates of blood transfusion (7.7% vs 20.1%) and marginally less complications (1.9% vs 2.6%). Those patients who received 2 g or more of TXA had a lower odds ratio of requiring a transfusion compared with those who received 1 g or less (0.31 vs 0.38).

Oremus and colleagues [8] used a randomised controlled study of 98 patients to conclude that patients were less likely to require an autologous blood transfusion if they received 1 g TXA on induction, with a further dose 3hours later. They also found that external blood loss was reduced with the use of TXA.

While there was no significant difference in functional improvement, measurements were taken one year apart. This temporal relationship introduces many variables and may have been more useful if functional scores were recorded in the first week postoperatively.

The rare occurrence of thromboembolic events in the study, compounded with the relatively low background incidence of clinically significant VTE, meant that no statistically significant observations regarding safety were established. Other studies published to date have been able to demonstrate that TXA is safe [14–17]. In particular, a large series of 822 patients assessing the use of TXA in cardiac surgery found no increased risk of thromboembolic events [14].

There were several limitations to this study. Firstly, the sample size was smaller than expected due to incomplete data recordings by theatre staff and progression to general anaesthesia without a spinal anaesthetic. These patients were excluded following randomisation and the procedure. Secondly, when transfusion rate is used as an outcome measure, it introduces some bias due to the variation in clinical triggers for transfusion from clinician to clinician. However, we aimed to assess transfusion rate based on the current management with an intention to treat analysis. Thirdly, we used a standardised dose of 1 g IV TXA, whereas the manufacturer's recommended dose is 15 mg/kg every eight hours over a 24-hour period [17]. Given a mean BMI of our patients of 28.9, we may have underdosed the sample on average, and therefore may have biased our results towards the null hypothesis.

## 5. Conclusion

The current literature suggests that TXA reduces postoperative blood loss and requirement for transfusion; however, this study did not provide any evidence to support this. Further studies are required to confirm the relationship between TXA and rate of transfusion and blood loss. They also need to establish the duration and dose of TXA appropriate for the type of surgery being performed. Focus is required on measuring outcomes of clinical importance such as cost and time to discharge. The safety of TXA in our general population should be confirmed, including in those who have had previous thromboembolic events.

## Figures and Tables

**Table 1:** Comparison of intraoperative blood loss and transfusion rates between TXA and placebo.

	TXA	Placebo	*p* value
Transfusion rate (units/patient)	0.34 (0–3)	0.44 (0–8)	0.70
Number of patients requiring transfusion (*n*/*N*, %)	8/42 (19.0%)	8/39 (20.5%)	1.00
Mean perioperative blood loss (mL), mean (range)	536.5 (130–920)	469.8 (57–1359)	0.28
Mean total blood loss in drains (mL), mean (range)	560.9 (0–1860)	688.4 (0–2500)	0.22
Day 1 haemoglobin levels (g/L), mean (range)	108.9 (82–132)	104.3 (80–143)	0.11
Day 4 haemoglobin levels (g/L), mean (range)	105.0 (83–129)	99.8 (75–136)	0.13
Time to discharge (days), mean (range)	4.3 (2.0–7.0)	4.8 (2.0–21.0)	0.20

## Data Availability

The data used to support the findings of this study are available from the corresponding author upon request.
